# Effect of automated unit dose dispensing with barcode scanning on medication administration errors: an uncontrolled before-and-after study

**DOI:** 10.1093/intqhc/mzab142

**Published:** 2021-10-18

**Authors:** Janique Gabriëlle Jessurun, Nicole Geertruida Maria Hunfeld, Joost Van Rosmalen, Monique Van Dijk, Patricia Maria Lucia Adriana Van Den Bemt

**Affiliations:** Department of Hospital Pharmacy, Erasmus MC, University Medical Center Rotterdam, P.O. Box 2040, Rotterdam, CA 3000, The Netherlands; Department of Hospital Pharmacy, Erasmus MC, University Medical Center Rotterdam, P.O. Box 2040, Rotterdam, CA 3000, The Netherlands; Department of Intensive Care, Erasmus MC, University Medical Center Rotterdam, P.O. Box 2040, Rotterdam, CA 3000, The Netherlands; Department of Biostatistics, Erasmus MC, University Medical Center Rotterdam, P.O. Box 2040, Rotterdam, CA 3000, The Netherlands; Department of Epidemiology, Erasmus MC, University Medical Center Rotterdam, P.O. Box 2040, Rotterdam, CA 3000, The Netherlands; Department of Internal Medicine, Section of Nursing Science, Erasmus MC, University Medical Center Rotterdam, P.O. Box 2040, Rotterdam, CA 3000, The Netherlands; Department of Hospital Pharmacy, Erasmus MC, University Medical Center Rotterdam, P.O. Box 2040, Rotterdam, CA 3000, The Netherlands; Department of Clinical Pharmacy and Pharmacology, University Medical Center Groningen, P.O. Box 30.001, Groningen, RB 9700, The Netherlands

**Keywords:** medication errors, patient safety, medication administration error, barcode, medication systems, hospital

## Abstract

**Background:**

Medication administration errors (MAEs) occur frequently in hospitals and may compromise patient safety. Preventive strategies are needed to reduce the risk of MAEs.

**Objective:**

The primary aim of this study was to assess the effect of central automated unit dose dispensing with barcode-assisted medication administration on the prevalence of MAEs. Secondary aims were to assess the effect on the type and potential severity of MAEs. Furthermore, compliance with procedures regarding scanning of patient and medication barcodes and nursing staff satisfaction with the medication administration system were assessed.

**Methods:**

We performed a prospective uncontrolled before-and-after study in six clinical wards in a Dutch university hospital from 2018 to 2020. MAE data were collected by observation. The primary outcome was the proportion of medication administrations with one or more MAEs. Secondary outcomes were the type and potential severity of MAEs, rates of compliance with patient identification and signing of administered medication by scanning and nursing staff satisfaction with the medication administration system. Multivariable mixed-effects logistic regression analyses were used for the primary outcome to adjust for confounding and for clustering on nurse and patient level.

**Results:**

One or more MAEs occurred in 291 of 1490 administrations (19.5%) pre-intervention and in 258 of 1630 administrations (15.8%) post-intervention (adjusted odds ratio 0.70, 95% confidence interval 0.51–0.96). The rate of omission fell from 4.6% to 2.0% and of wrong dose from 3.8% to 2.1%, whereas rates of other MAE types were similar. The rate of potentially harmful MAEs fell from 3.0% (*n* = 44) to 0.3% (*n* = 5). The rates of compliance with scanning of patient and medication barcode post-intervention were 13.6% and 55.9%, respectively.

The median overall satisfaction score of the nurses with the medication administration system on a 100-point scale was 70 (interquartile range 63–75, *n* = 193) pre-intervention and 70 (interquartile range 60–78, *n* = 145) post-intervention (*P *= 0.626, Mann–Whitney *U* test).

**Conclusion:**

The implementation of central automated unit dose dispensing with barcode-assisted medication administration was associated with a lower probability of MAEs, including potentially harmful errors, but more compliance with scanning procedures is needed. Nurses were moderately satisfied with the medication administration system, both before and after implementation. In conclusion, despite low compliance with scanning procedures, this study shows that this intervention contributes to the improvement of medication safety in hospitals.

## Introduction

A recent meta-analysis has shown that at least 1 in 20 patients is affected by preventable patient harm in healthcare settings and that approximately 12% of preventable harm causes permanent disability or patient death [1]. Medication-related incidents account for the highest proportion of preventable harm [1]. Thus, although drug therapy remains a cornerstone for the treatment of many diseases, possible process difficulties may compromise patient safety. Medication administration errors (MAEs) occur in about 10%, ranging from approximately 5% to 20% of medication administrations in hospitals [2, 3]. Many interventions to prevent these errors have been implemented [4–9]. However, the remaining high MAE rates warrant additional system defences.

Promising interventions include automated unit dose dispensing (ADD) and barcode-assisted medication administration (BCMA) [4–6, 9–13]. Combining central ADD with BCMA may have a synergistic effect on medication errors by facilitating a closed-loop system, when combined with an electronic medical record (EMR) or a computerized physician order entry (CPOE) system. Studies on central ADD have shown relative reductions in MAE rates of approximately 50–60% [14, 15], while most studies on the effect of BCMA have shown relative reductions of 30–60% [9, 12]. So far, only two studies, performed in acute medical or haematological wards, have examined the combined effect of central ADD with BCMA on MAEs, with CPOE already in place [16, 17]. These studies have shown relative MAE reduction values of 62% [16] and 94% [17], respectively.

As only limited evidence is available, we performed a before-and-after study aimed to assess the effect of central ADD with BCMA on the prevalence, type and severity of MAEs; compliance with patient identification and signing of administered medication by scanning; and nursing staff satisfaction with the medication administration system.

## Methods

### Study design

We performed a prospective before-and-after study in Erasmus MC, University Medical Center Rotterdam in Rotterdam, the Netherlands. The local Medical Ethics Review Committee waived approval for this study (reference number MEC-2018-1532) in accordance with the Dutch Medical Research Involving Human Subjects Act. Nursing staff gave verbal informed consent to participate in this study. Data were handled confidentially according to the Dutch General Data Protection Regulation. The Strengthening the Reporting of Observational Studies in Epidemiology Statement guideline was used for reporting (Supplementary File 1) [18].

### Study setting

The study was performed in six clinical wards (internal oncology, neurology, pulmonary medicine, haematology, neurosurgery and hepatopancreatobiliary surgery). Pre-intervention, an EMR system, a CPOE system and an electronic medication administration record (eMAR) system were in place using HiX® software (ChipSoft B.V., Amsterdam, the Netherlands). An additional CPOE system, Practocol® (Practocol B.V., Rotterdam, the Netherlands), was used for medication prescription and administration in chemotherapy protocols.

The intervention consisted of central ADD (in the hospital pharmacy) and BCMA, which were integrated in HiX®. ADD and BCMA were gradually implemented in the entire hospital, starting mid-2019. Detailed setting characteristics are shown in Table 1.

**Table 1:** Setting characteristics before and after the implementation of central automated unit dose dispensing and barcode-assisted medication administration

Characteristics	Pre-intervention	Post-intervention
EMR system^a^	HiX®	HiX®
CPOE system^a^	HiX®, Practocol®	HiX®
Central ADD^a^	Not applicable	– Pillpick® in the central hospital pharmacy – Yes, for oral medication primarily – Automated processing of prescriptions in CPOE – Barcoded unit dose plastic bags (with medication name, strength, expiry date, lot number and national article identifier) attached to a plastic ring (supply for 12–24 h). General information of patient and attached medication (name, strength and administration time) are printed and attached to the ring. – Automated checking of expiry dates
Medication supply	– By the central hospital pharmacy – First order: ordered by pharmacy technicians when processing prescriptions in HiX® – Follow-up orders: by nursing staff electronically in HiX®. Telephone orders possible. – Multidose preparations such as inhalers: on request.	– Prescriptions automatically processed by the ADD software – Other medication: as pre-intervention
Medication stocking	Ward-based stock (tailored): emergency medication, commonly used medication and patient-specific medication (for several days)	As pre-intervention, but a smaller range of commonly used medication
Medication cart filling^b^	By nurses, generally for 24 h	– By nurses, generally for 24 h—three wards – By pharmacy staff centrally for 24 h (weekdays) or 72 h (Friday)—three wards
BCMA		
Patient identification by scanning	Possible, but not standard practice	Yes
Medication identification by scanning	No	Yes
BCMA features of medication identification^a^	Not applicable	– Visual alerts in eMAR HiX® – Wrong medication, strength per unit, dosage form – No automated dose checking (e.g. number of tablets)
Workstations	On mobile medication carts with scanners	On mobile medication carts with scanners
Use of patient’s own medication or self-administration	Not standard practice, only under strict protocols	Not standard practice, only under strict protocols
Signing of administered medication^a^	In eMAR HiX®: manually by nursing staff	In eMAR HiX®: manually or by scanning medication by nursing staff

a HiX® version 6.1 (ChipSoft B.V.; Amsterdam, the Netherlands); Practocol® version 2.0.9.3 and 2.1.5.1 (Practocol B.V.; Rotterdam, the Netherlands) for medication in chemotherapy protocols (e.g. dexamethasone); Pillpick® (Swisslog; Buchs, Switzerland).

b Procedures differed between wards because central filling was hampered by limited human resources.

### Inclusion and exclusion criteria

Medication administrations to patients performed by nursing staff were included. Excluded were medication administrations that were terminated during the observation, medication administrations with the medication name missing on the data collection form or medication administrations that were declined by patients for other reasons than being erroneous.

### Data collection

Data were collected from October 2018 to February 2019 pre-intervention and from September 2020 to December 2020 post-intervention. All post-intervention measurements took place at least 3 months after complete implementation. Data on medication administration were collected using the disguised observation method [19–21], meaning that nursing staff did not know the exact purpose of the observations. Nursing staff were informed that the purpose of the observations was to study the medication process to minimize the effect of the observer on the observed (Hawthorne effect). Eight observers, one pharmacist and seven students with a pharmaceutical or medical background, that had completed a training programme of several days shadowed nursing staff and recorded details of every medication administration on data collection forms. Observers used the convenience sampling method to select nurses to be observed when present in the ward and asked them for verbal consent before initiating an observation. Observers were instructed to intervene if they perceived a serious error to be occurring (wrong patient, wrong drug or a dose deviation of at least 20%). Data collected during observation were compared with medication prescriptions and protocols after the observation and not during observation, which is in accordance with the gold standard of medication error detection methods [20]. Initially, one pharmacist (J.J.) and hospital pharmacist (N.H.) independently reviewed 200 data collection forms to assess the presence, type and potential severity of MAEs. For this sample, the Cohen’s kappa for the presence of one or more MAE was calculated at 0.72, which indicates substantial interrater reliability. Therefore, the remaining data collection forms were reviewed by one reviewer (J.J.) to determine the presence of an MAE. The type and potential severity of each MAE were classified by J.J. and N.H. based on the literature and experience, while disagreement was resolved by consensus.

Nurses were asked to fill in a questionnaire to collect data on their background characteristics (i.e. gender, age, degree type, educational level, experience and employment type) after the completion of observation rounds in a particular ward. For each observed medication administration, J.J. assessed the day of the week and whether the medication was signed as administered in the eMAR (signed as administered in HiX®: yes, no; scanned medication barcode according to HiX®: yes, no). Patient-characteristic data (i.e. gender, birth date and number of prescribed medications) were collected by J.J. from Practocol® and HiX®.

Collected data of the medication administrations were entered in OpenClinica® version 2.1 (OpenClinica LLC, Waltham, Massachusetts, USA).

#### Nursing staff satisfaction questionnaire

The *Medication Administration System—Nurses Assessment of Satisfaction (MAS-NAS) scale* [22] was translated to Dutch. This questionnaire was presented twice to the nursing staff. Pre-intervention, MAS-NAS measured the satisfaction with the medication administration system without ADD-BCMA and post-intervention, it measured the satisfaction of the system with ADD-BCMA. The questionnaire consisted of a question concerning overall satisfaction, 15 statements (on efficacy, safety and access) and an open-ended question. Nurses indicated overall satisfaction on a visual analogue scale from 0 (*dissatisfied*) to 100 (*satisfied*). Responses on the 15 statements were given on a 6-point Likert scale from 1 (*strongly disagree*) to 6 (*strongly agree*). The open-ended question invited additional remarks. Trained students visited the clinical wards and presented the questionnaire on an iPad® (Apple Inc.; Cupertino, California, USA) to the nursing staff present.

### Definition and classification of MAE

An MAE was defined as a deviation from medication orders used by the nursing staff to administer medication; a deviation from general medication administration protocols; and if local protocols were not available, a deviation from the medication information sheet provided by the manufacturer. Timing errors were not within the scope of the study because they are generally considered not to be clinically relevant [2, 3]. Intravenous admixture preparation errors, such as wrong solvents and hygiene errors, as well as procedural errors, such as labelling and documentation errors, were not considered as MAEs.

MAEs were classified into the following types [19, 23]: wrong administration technique (too fast administration, incompatibility of parenteral medication and other), wrong medication handling (e.g. not shaking suspensions, wrong infusion fluid/infusion fluid volume or combining medication solutions), omission, wrong dose, unordered drug, wrong dosage form, wrong route of administration, expired medication and other. Omissions were defined as medication not administered during the day of observation. This was the case if all of the following conditions were met: (i) the observer observed the entire medication round for the patient (e.g. of 8 a.m.), (ii) the nurse did not administer the medication and (iii) the medication was not signed as administered in the eMAR by another nurse or outside the observed period. For wrong dose, too fast administration and wrong infusion fluid volume, a deviation of more than 10% were marked incorrect because a maximum of 10% deviation from the declared dose of pharmaceutical products within the shelf life is widely accepted (e.g. by manufacturing guidelines). For the classification of the potential severity of MAEs, the National Coordinating Council for Medication Error Reporting and Prevention (NCC MERP) severity index was used, which ranges from category A (circumstances that have the capacity to cause error) to I (death) [24]. Errors classified in NCC MERP class E or higher were considered potentially harmful.

### Outcomes

The primary outcome was the proportion of medication administrations with one or more MAEs using the total opportunities for error as the denominator (i.e. the number of observed administrations plus the identified omissions). Secondary outcomes were the type and potential severity of MAEs, rates of compliance with patient identification by scanning and electronic signing of administered medication by scanning, and nursing staff satisfaction with the medication administration system.

### Sample size calculation

To identify a reduction in MAEs from 10% to 5% [2, 3, 10, 12, 25], a sample size of 868 medication administrations both pre-intervention and post-intervention would be required, based on a two-sided chi-square test using α of 0.05 and β of 0.2. The same dataset was planned to be used for a study on potential risk factors for MAEs, for which sample sizes of 2000 pre-intervention and 4000 post-intervention were calculated. Therefore, this larger number of medication administrations was pursued to be included. Ninety-six observation rounds in each measurement period were planned beforehand, distributed over different time windows and days of the week.

### Data analysis

MAE rates were compared using univariable and multivariable mixed-effects logistic regression analysis (generalized linear mixed models). The dependent variable in these models was a dichotomous variable indicating whether a medication administration had one or more MAEs (yes, no); and the independent variables were setting (pre-intervention and post-intervention) and the covariates pharmaceutical form (oral solid, oral liquid, infusion, injection, nebulizing solution, ointment, suppository/enema and miscellaneous), time window of administration (7 a.m.–10 a.m., 10 a.m.–2 p.m., 2 p.m.–6 p.m. and 6 p.m.–7 a.m.), clinical ward type, nursing degree type (nurse, specialised nurse and other) and nursing educational level (secondary vocational education, higher professional education and other). A model with and without nurse characteristics was presented because data on nurse characteristics were only available for 189 of 359 observed nurses (52.6%). We included two random effects, i.e. a random intercept by staff member and a random intercept by patient to account for repeated measurements and within-subject correlations. Complete case analyses were performed. The results of the mixed-effects logistic regression analyses are reported as adjusted odds ratios (ORadj) with 95% confidence intervals (95% CIs).

The Mann–Whitney *U* test was used to compare the overall nursing staff satisfaction scores, which were not normally distributed. Descriptive statistics were used for other secondary outcomes. A two-tailed *P* value <0.05 was considered statistically significant. Data analyses were performed with R Statistics® version 4.0.2. (The R Foundation, Vienna, Austria) for the mixed-effects logistic regression analyses and with SPSS Statistics^®^ version 25 (IBM Corporation, Armonk, New York, USA) for other analyses.

## Results

A total of 3191 medication administrations administered were observed. Seventy-one observations were excluded because patients declined administration. Observers intervened in six administrations pre-intervention (omission, *n* = 1; wrong dose, *n* = 2; unordered drug [wrong patient], *n* = 3) and in one administration post-intervention (omission, *n* = 1). The characteristics of the included 3120 medication administrations are shown in Table 2.

**Table 2:** Characteristics of included medication administrations before and after implementation of central automated unit dose dispensing and barcode-assisted medication administration

Characteristics	Pre-intervention *N* = 1490 medication administrations	Post-intervention *N* = 1630 medication administrations
*Patient characteristics*		
Patients, *n*	245	253
Male, *n* (% of patients)	145 (59.2)	128 (50.6)
Age, median (IQR)	62 (50–70)	61 (47–69)
Prescribed medications per day, median (IQR)	13 (10–16)	13 (10–17)
*Medication characteristics*		
Pharmaceutical form^a^, *n* (% of administrations)		
Oral solid	936 (62.8)	1021 (62.6)
Oral liquid	66 (4.4)	87 (5.3)
Infusion	252 (16.9)	239 (14.7)
Injection	136 (9.1)	202 (12.4)
Nebulizing solution	47 (3.2)	35 (2.1)
Ointment	10 (0.7)	11 (0.7)
Suppository/enema	17 (1.1)	8 (0.5)
Miscellaneous^b^	25 (1.7)	26 (1.6)
*Ward characteristics*		
Clinical ward, *n* (% of administrations)		
Internal oncology	252 (16.9)	285 (17.5)
Neurology	196 (13.2)	218 (13.4)
Pulmonary medicine	375 (25.2)	278 (17.1)
Haematology	234 (15.7)	215 (13.2)
Neurosurgery	281 (18.9)	351 (21.5)
Hepatopancreatobiliary surgery	152 (10.2)	283 (17.4)
*Time characteristics*		
Day of the week, *n* (% of administrations)		
Weekday	985 (66.1)	1097 (67.3)
Weekend	505 (33.9)	533 (32.7)
Time of administration, *n* (% of administrations)		
7 a.m.–10 a.m.	454 (30.5)	497 (30.5)
10 a.m.–2 p.m.	236 (15.8)	248 (15.2)
2 p.m.–6 p.m.	273 (18.3)	335 (20.6)
6 p.m.–7 a.m.	527 (35.4)	550 (33.7)
*Workload characteristics*		
Patient-to-nurse ratio^c^, median (IQR)	5 (4–7)	5 (4–7)
Interruptions		
Yes	96 (6.4)	70 (4.3)
*Staff characteristics*		
Observed staff members, *n*	179	180
Staff members, personal data available, *n* (% of staff)	107 (59.8)	82 (45.6)
Male, *n* (% of staff)	7 (6.5)	6 (7.3)
Age^d^, median (IQR)	30 (25–50)	27 (23–35)
Degree type, *n* (% of staff)		
Nurse	68 (63.6)	53 (64.6)
Specialized nurse	27 (25.2)	18 (22.0)
Student nurse	10 (9.3)	10 (12.2)
Other	2 (1.9)	1 (1.2)
Educational level^e^, *n* (% of staff)		
Secondary vocational education	46 (43.4)	40 (48.8)
Higher professional education	49 (46.2)	42 (51.2)
University education	1 (0.9)	0
Other	10 (9.4)	0
Experience since nursing diploma, *n* (% of staff)		
0–1 year	18 (16.8)	11 (13.4)
1–5 years	20 (18.7)	33 (40.2)
More than 5 years	60 (56.1)	27 (32.9)
Not applicable	11 (10.3)	11 (13.4)
Experience in healthcare settings^e^, *n* (% of staff)		
0–1 year	1 (0.9)	3 (3.7)
1–5 years	34 (32.1)	32 (39.0)
More than 5 years	71 (67.0)	47 (57.3)
Employment type^e^, *n* (% of staff)		
Non-temporary	97 (91.5)	74 (90.2)
Temporary	6 (5.7)	8 (9.8)
Other	3 (2.8)	0

IQR, interquartile range.

a Missing, *n* = 1 (pre-intervention), *n* = 1 (post-intervention). ^b^Miscellaneous: inhalers, patches, eye drops/ointments, intestinal gel. ^c^Missing, *n* = 61 (pre-intervention), *n* = 128 (post-intervention). ^d^Missing, *n* = 7 (post-intervention). ^e^Missing, *n* = 1 (pre-intervention).

### Prevalence, type and potential severity of MAEs

One or more MAEs were identified in 291 of 1490 administrations (19.5%) pre-intervention and in 258 of 1630 administrations (15.8%) post-intervention (ORadj 0.70, 95% CI 0.51–0.96; Table 3). A total of 316 MAEs and 272 MAEs occurred, respectively, before and after implementation of the intervention.

**Table 3:** Effect of central automated unit dose dispensing and barcode-assisted medication administration on medication administration errors (MAEs)

		Mixed-effects logistic regression analysis^a^		
	MAE prevalence	Univariable analysis *N* = 3097	Multivariable analysis^b^ *N* = 3095	Multivariable analysis with nurse characteristics^c^ *N* = 1561
*Measurement period*	*n/N (%)*	*OR (95% CI)*	*Adjusted OR (95% CI)*	*Adjusted OR (95% CI)*
Pre-intervention	291/1490 (19.5)	Reference	Reference	Reference
Post-intervention	258/1630 (15.8)	0.76 (0.55–1.04)	0.70 (0.51–0.96)*	0.57 (0.37–0.88)*

CI, confidence interval; OR, odds ratio.

a Mixed-effects logistic regression analysis was used to account for within-subject correlations due to repeated measurements by staff member and patient.

b ORs have been adjusted for pharmaceutical form, time window and clinical ward type.

c ORs have been adjusted for nurse educational level, nurse degree type, pharmaceutical form, time window and clinical ward type.

* Statistically significant (*P* < 0.05).

The type and potential severity of identified MAEs are shown in Table 4. After the intervention, the rate of omission fell from 4.6% to 2.0% and of wrong dose from 3.8% to 2.1%, while rates of other MAE types were similar. The prevalence of potentially harmful MAEs fell from 3.0% (*n* = 44) to 0.3% (*n* = 5). Examples of the potential severity of MAEs are described in Supplementary File 1.

**Table 4:** Type and potential severity of medication administration errors (MAEs) before and after implementation of central automated unit dose dispensing and barcode-assisted medication administration

	Pre-intervention	Post-intervention
*Included medication administrations, n*	*1490*	*1630*
*MAEs, n*	316	272
*Type of MAE, n (% of administrations)*		
Wrong administration technique	78	99
Too fast administration	51 (3.4)	83 (5.1)
Incompatibility of parenteral medication	21 (1.4)	3 (0.2)
Other	6 (0.4)	13 (0.8)
Wrong medication handling	57 (3.8)	59 (3.6)
Omission	68 (4.6)	33 (2.0)
Wrong dose	57 (3.8)	35 (2.1)
Unordered drug	25 (1.7)	26 (1.6)
Wrong dosage form	25 (1.7)	20 (1.2)
Wrong route of administration	5 (0.3)	0
Expired medication	0	0
Other	1 (0.1)	0
*Potential severity of MAEs*^a^, *n* *(% of administrations)*		
Error, no harm		
C	173 (11.6)	209 (12.8)
D	99 (6.6)	58 (3.6)
Error, harm		
E	35 (2.3)	5 (0.3)
F	7 (0.5)	0
H	2 (0.1)	0

a NCC MERP classification [24]: no error (category A); error, no harm (category B to D); error, harm (category E to H); and error, death (category I). C: an error occurred that reached the patient but did not cause patient harm; D: an error occurred that reached the patient and required monitoring to confirm that it resulted in no harm to the patient and/or required intervention to preclude harm; E: an error occurred that may have contributed to or resulted in temporary harm to the patient and required intervention; F: an error occurred that may have contributed to or resulted in temporary harm to the patient and required initial or prolonged hospitalization; H: an error occurred that required intervention necessary to sustain life.

### Compliance with patient identification and signing of administered medication

Table 5 shows the rates of compliance with procedures regarding patient identification and electronic signing of administered medication. Barcode scanning for patient identification was performed in 124 of 1490 administrations (8.3%) pre-intervention and in 221 of 1630 administrations (13.6%) post-intervention. Electronic signing of administered medication was performed in 1418 administrations (95.2%) pre-intervention and in 1575 administrations (96.6%) post-intervention. Post-intervention, medication barcodes were scanned in 911 administrations (55.9%).

**Table 5:** Rates of compliance with patient identification and electronic signing of administered medication before and after the implementation of central automated unit dose dispensing and barcode-assisted medication administration

	Pre-intervention *N* = 1490	Post-intervention *N* = 1630
Procedures	*Rate, n (% of N)*	*Rate, n (% of N)*
*Patient identification*		
By barcode scanning		
Yes	124 (8.3)	221 (13.6)
No	1251 (84.0)	1300 (79.8)
Unknown	115 (7.7)	109 (6.7)
*Signing of administered medication*		
Signed in eMAR		
Yes	1418 (95.2)	1575 (96.6)
No	71 (4.8)	55 (3.4)
Unknown	1 (0.1)	0
By barcode scanning		
Yes	Not applicable	911 (55.9)
No	Not applicable	664 (40.7)

eMAR, electronic medication administration record.

### Post hoc analysis: scanned versus non-scanned medication (post-intervention)

A post hoc analysis showed that MAE rates were 13.0% (*n*/*N* = 118/911) for scanned medication and 19.5% (*n*/*N* = 140/719) for non-scanned medication. The rates of the following MAE types were lower for scanned medication compared to non-scanned medication: omission (*n* = 3 versus *n* = 30; 0.3% versus 4.2%), unordered drug (*n* = 6 versus *n* = 20; 0.7% versus 2.8%), wrong dosage form (*n* = 3 versus *n* = 17; 0.3% versus 2.4%) and wrong dose (*n* = 14 versus *n* = 21; 1.5% versus 2.9%). The rates of the following MAE types were higher or similar for scanned medication compared to non-scanned medication: wrong administration technique (*n* = 64 versus *n* = 35; 7.0% versus 4.9%) and wrong medication handling (*n* = 32 versus *n* = 27; 3.5% versus 3.8%). All potentially harmful MAEs (*n* = 5) occurred with non-scanned medication.

### Nursing staff satisfaction with the medication administration system

The median overall satisfaction score with the medication administration system on a 100-point scale was 70 (interquartile range 63–75, *n* = 193) pre-intervention and 70 (interquartile range 60–78, *n* = 145) post-intervention (*P *= 0.626). Median satisfaction scores on a 6-point scale with regard to 15 statements were moderate (score 3) to high (score 5) (Supplementary File 2). Nurses were moderately satisfied with topics regarding safety related to MAEs, timeliness of acute medication availability, facilitation of communication, information in case of adverse reactions, information on medication actions and adverse effects (post-intervention) and the necessity to hoard medication (pre-intervention). Before intervention, remarks were particularly related to optimizing dispensing times. After intervention, remarks were primarily related to technical issues with scanning (e.g. slow response time) and shortfalls of the system to check the right dose (e.g. half tablets).

## Discussion

### Statement of principal findings

The implementation of central ADD with BCMA reduced the probability of medication errors during administration from 19.5% to 15.8% and of potentially harmful errors from 3.0% to 0.3%. Procedures for patient identification and signing of administered medication by scanning were not fully adhered to. In the post-implementation period, error rates were lower for scanned medication compared to non-scanned medication (13.0% versus 19.5%) and all potentially harmful errors occurred with non-scanned medication. Compared to non-scanned medication, scanned medication had lower rates of all error types, i.e. omission, unordered drug, wrong dosage form and wrong dose, except for wrong administration technique and wrong medication handling. Overall, nursing staff satisfaction with the medication administration system was moderate and did not change after implementing the intervention.

### Interpretation within the context of the wider literature

The MAE frequency reduction found in this study is in line with previous studies examining the effect of central ADD, BCMA and/or closed-loop systems [4, 6, 9, 10, 12–17, 25]. Findings of studies on closed-loop systems are difficult to compare because of heterogeneity with regard to studied interventions (e.g. solely BCMA), baseline setting characteristics (e.g. paper-based systems versus electronic systems) and patient populations. To our knowledge, only two Danish studies have examined the effect of the combined intervention with EMR and CPOE systems already in place [16, 17]. These controlled before-and-after studies have shown a higher reduction of MAEs (odds ratio 0.38 [16] and 0.06 [17]). However, it is difficult to compare these studies with our study because they were performed in acute medical wards or haematological wards, using non-disguised observational methods and focusing solely on oral medication [16, 17]. Other studies on central ADD have shown reductions in MAE rates of approximately 50–60% [14, 15], while most studies on BCMA have shown a reduction of 30–60% after its implementation [9, 12]. However, the absolute MAE reduction found in this study (3.7%) is quite comparable to that in previous studies on cADD or BCMA [9, 12, 14, 15].

The rate of omission reduced from 4.6% to 2.0% and of wrong dose from 3.8% to 2.1%, while rates of other MAE types were unaffected. Comparing scanned versus non-scanned medication in the post-implementation period also showed varying effects on different error types. This supports the claim that the examined intervention only has the ability to reduce specific types of errors [9, 10, 12, 13].

Limited compliance with patient and medication identification by scanning may have diluted the positive effect of the intervention in our study. Potential causes of non-compliance may include technical difficulties, time constraints and incomplete integration in the standard workflow [26]. Shortcomings in implementation, design and workflow integration are known triggers of workarounds [27], which subsequently may lead to reduced safety effects [10, 27–29]. Workarounds observed in our study include affixing patient identification barcodes to beds and carrying several patients’ pre-scanned medication on carts. Other issues observed include difficulties while using the scanner (e.g. slow response), insufficient number of scanners or medication carts and non-barcoded medication.

### Strengths and limitations

Strength of this study is that we included a large number of representative medication administrations performed by many staff members of different clinical wards, supporting the generalizability of our results to similar settings. Another strength is that we used a robust method to identify and assess MAEs in daily clinical practice. This study also has some limitations. First, observer bias may have occurred. We tried to limit this by using the disguised method [19–21] and obligatory extensive training programmes for observers. Second, a potential limitation of before-and-after studies is that other changes in the medication process (e.g. other patient safety initiatives) may have influenced the results. However, to our knowledge, no additional substantial changes related to the medication administration process were made. Finally, the monocentre setting may limit generalizability.

### Implications for policy, practice and research

The findings of this study support the implementation of central ADD with BCMA. However, this study also emphasizes the need for comprehensive implementation strategies and ongoing evaluation strategies (e.g. by using the Plan-Do-Study-Act method [30]). Scanning procedures were not fully adhered to, although extensive resources were expended for the implementation in our institution. Exploration of facilitators and barriers for implementation of such interventions seems crucial because not using patient-safety technology as intended may compromise the efficacy of such interventions [10, 26–28]. Also, such interventions should be co-developed with all stakeholders, especially the target audience, to tailor the technology to the needs of the people that will be using it.

## Conclusions

Central ADD with BCMA was associated with a reduced frequency of MAEs, including potentially harmful errors, but compliance with scanning patient and medication barcodes needs improvement. In conclusion, this study shows that this intervention contributes to the improvement of medication safety in hospitals.

## Supplementary Material

mzab142_Supp

## Data Availability

The data underlying this article will be shared on reasonable request to the corresponding author.
