# Supplementary material for: Effect of automated unit dose dispensing with barcode scanning on medication administration errors: an uncontrolled before-and-after study
Source: Int J Qual Health Care. 2021 Oct 18;33(4):mzab142. doi: 10.1093/intqhc/mzab142 (PMC8678992; doi:10.1093/intqhc/mzab142)
Supplement: mzab142_Supp [file mzab142_supp.zip › 20211029 Supplementary file 2_Potential severity of errors.docx]

**Effect of automated unit dose dispensing with barcode scanning on medication administration errors: an uncontrolled before-and-after study**

**Supplementary file 2**

*Examples of the potential severity of different medication administration errors*

| ***NCC MERP category*** ^1^ | ***Example*** |
| --- | --- |
| ***C*** | - Too fast administration of dexamethasone or granisetron (in a few seconds) - Omission of dermal menthol cream - Wrong dosage form of acetaminophen (e.g. suppository administered rectally instead of tablet administered orally) |
| ***D*** | - Oral antacid suspension not shaken - Wrong dose of macrogol laxatives (1 sachet instead of 2 sachets) - Wrong dose of oral esomeprazole (20 mg instead of 40 mg) |
| ***E*** | - Wrong administration rate of intravenous fentantyl (2 ml/h instead of 4 ml/h) - Wrong dose of intravenous lorazepam (4 mg instead of 1 mg) - Unordered drug: oral diazepam 10 mg |
| ***F*** | - Omission of oral valproic acid 1000 mg - Wrong dose of intravenous colistin (50000 IE instead of 500000 IE) |
| ***H*** | - Wrong dose of subcutaneous nadroparin (5700 IE instead of 11400 IE) in an oncological clinical ward - Wrong dose of subcutaneous nadroparin (9500 IE instead of 19000 IE) in an oncological clinical ward |

^1^ NCC MERP categorisation: no error (category A); error, no harm (category B to D); error, harm (category E to H); and error, death (category I). C: an error occurred that reached the patient but did not cause patient harm; D: an error occurred that reached the patient and required monitoring to confirm it resulted in no harm to the patient and/or required intervention to preclude harm; E: an error occurred that may have contributed to or resulted in temporary harm to the patient and required intervention; F: an error occurred that may have contributed to or resulted in temporary harm to the patient and required initial or prolonged hospitalization; H: an error occurred that required intervention necessary to sustain life [1]

[1] Snyder RA, Abarca J, Meza JL, Rothschild JM, Rizos A, Bates DW. Reliability evaluation of the adapted national coordinating council medication error reporting and prevention (NCC MERP) index. *Pharmacoepidemiol Drug Saf*. 2007; 16: 1006-13.
