# Supplementary material for: Effect of automated unit dose dispensing with barcode scanning on medication administration errors: an uncontrolled before-and-after study
Source: Int J Qual Health Care. 2021 Oct 18;33(4):mzab142. doi: 10.1093/intqhc/mzab142 (PMC8678992; doi:10.1093/intqhc/mzab142)
Supplement: mzab142_Supp [file mzab142_supp.zip › 20211029 Supplementary file 3_Questionnaire scores.docx]

**Effect of automated unit dose dispensing with barcode scanning on medication administration errors: an uncontrolled before-and-after study**

**Supplementary file 3**

*Nursing staff satisfaction with the medication administration system before and after implementation of central automated dose dispensing and barcode-assisted medication administration using The Medication Administration System--Nurses Assessment of Satisfaction (MAS-NAS) scale*

| ***Nursing staff satisfaction*** | ***Pre-intervention*** | | ***Post-intervention*** | |
| --- | --- | --- | --- | --- |
|  | *n* | *Score* | *n* | *Score* |
| Sub-questions (1 Strongly disagree - 6 Strongly agree), median (IQR)  1 Efficiency  2 User friendliness  3 Access to materials/necessities  4 Safety in relation to medication administration errors  5 Timeliness of acute medication availability  6 Access to active prescriptions  7 Information on pharmacist review of medication order  8 Ease to check the five medication administration rights  9 Facilitation of communication between doctor, nurse, and pharmacist  10 Access to general medication information  11 Access to information when adverse reactions occur  12 Information on medication actions and adverse effects  13 Necessity to hoard medication  14 Access to systems necessary for administering medication  15 Knowledge on storage locations | 195  195  195  195  194  194  193  194  193  194  194  194  193  193  195 | 5 (4-5)  5 (4-5)  4 (4-5)  4 (3-5)  3 (3-4)  5 (4-5)  4 (4-5)  4 (4-5)  4 (3-5)  5 (4-5)  4 (3-5)  4 (4-5)  4 (3-5)  5 (4-5)  5 (4-5) | 145  145  144  145  145  144  144  144  145  144  145  144  145  144  145 | 4 (4-5)  4 (4-5)  4 (4-5)  4 (3-5)  4 (3-5)  5 (4-5)  5 (4-5)  5 (4-5)  4 (3-5)  5 (4-5)  4 (3-5)  4 (3-5)  4 (4-5)  5 (4-5)  5 (4-5) |

IQR, interquartile range
